# Mitochondrial DNA phylogeography of a species-specific sucking louse, *Johnsonpthirus heliosciuri*, act as a proxy to provide insights into the population connectivity of its host, smith’s bush squirrels, *Paraxerus cepapi*

**DOI:** 10.1007/s00436-025-08594-x

**Published:** 2026-01-27

**Authors:** Inge Raubenheimer, Sonja Matthee, Jeanette Wentzel, Conrad A. Matthee

**Affiliations:** 1 Department of Conservation Ecology and Entomology, Stellenbosch University, Private Bag X1, Matieland, 7602 South Africa; 2 Department of Veterinary Tropical Diseases, University of Pretoria, Hatfield, South Africa; 3 Evolutionary Genomics Group, Department of Botany and Zoology, Stellenbosch University, Private Bag X1, Matieland, 7602 South Africa

**Keywords:** Anoplura, Co-evolution, Evolutionary history, Genetic structure, Permanent parasite, Sciuridae, south africa

## Abstract

Due to co-evolution, permanent species-specific lice and their hosts often exhibit congruent phylogenetic patterns, and to a lesser extent also congruent phylogeographic structures. However, because ectoparasites generally have smaller effective population sizes (due to their aggregated distribution), and they have a faster evolutionary rate (generation time effect), their phylogeographic structures are often more pronounced. This study investigates the phylogeographic structure of a squirrel *Paraxerus cepapi* and one of its louse species, *Johnsonpthirus heliosciuri*, sampled from eight localities in South Africa. Statistical haplotype networks derived from 51 host mitochondrial DNA control region sequences revealed a lack of geographic genetic structure among sampling sites, with most genetic variation found within populations (Φ_ST_ = 0.304, *P* < 0.05). In stark contrast, analyses of 43 louse mitochondrial COI sequences showed a clear pattern of geographic genetic structure, with most variation occurring between populations (Φ_ST_ = 0.797, *P* < 0.05). Nuclear Eukaryotic Elongation Factor 1 (EF1) data revealed no geographic structure in either species. The lack of phylogeographic congruence between host and louse, as well as between mitochondrial and nuclear markers, is likely due to stochastic differences in the evolutionary rates of host and parasite DNA. In this study, the species-specific permanent parasite acted as a biological proxy, or a ‘magnifying glass’, for host phylogeography. The louse mitochondrial DNA data suggest that recent anthropogenic habitat fragmentation may indeed be limiting squirrel movement across the landscape.

## Introduction

The comparison of genetic patterns between a host and its associated parasites can give insight into the evolutionary history of host taxa (Nieberding et al. 2004; Nieberding and Morand 2006; Whiteman et al. 2007; Light and Hafner 2008; du Toit et al. 2013; Bothma et al. 2021). Congruent evolutionary histories between a parasite and its host depend on micro-evolutionary (e.g., genetic drift, selection) and macro-evolutionary processes (e.g., cladogenesis) (Huyse et al. 2005), and these processes are generally correlated to host life history (e.g., dispersal ability), parasite life history (e.g., generalist or specialist), and off-host factors (e.g., time spent in the external environment) (Blouin et al. 1995; Nadler 1995; Johnson et al. 2003; Criscione and Blouin 2004). Although environmental conditions such as temperature and humidity often influence ectoparasites with free-living stages (such as ticks and fleas (Linardi and Krasnov 2012; Matthee et al. 2013; Van der Mescht et al. 2016; Wale et al. 2023) a permanent parasite, such as a species-specific louse, is less influenced by external environmental factors because they remain on the host where they are buffered by the host microclimate (Kim 2006; but see Moyer et al. 2002 and Bush et al. 2024). This can mainly be attributed to the fact that lice with a direct life cycle have no intermediate host and no free-living life stages, and their mode of dispersal is primarily through direct bodily contact among host individuals belonging to the same species and who occur in proximity (Barker 1994; du Toit et al. 2013; Durden and Lloyd 2019). In such systems, vertical transmission of the parasite (e.g., mother to offspring during nest sharing and grooming) is most likely to occur (Clayton et al. 2003). It is thus not unexpected that at least some phylogeographic congruence between lice and their hosts occurs (du Toit et al. 2013; Sweet et al. 2018; Bell et al. 2021; Bothma et al. 2021).

The loss of genetic diversity and the fixation rate of unique changes in the DNA of parasites are often faster when compared to their hosts because of the effects of genetic drift on small populations (Hafner et al. 1994; Nieberding et al. 2004; Light and Hafner 2008). For example, the preservation of haplotypic diversity is directly linked to the effective population size of the population, where aggregated parasite populations will experience a much higher level of stochasticity compared to host populations that are characterized by larger effective population sizes (Hague and Routman 2016). Furthermore, many parasites including lice can have higher mutation rates compared to their hosts (Bromham, 2009 and refs therein) resulting in genetic structure in the parasite before it becomes apparent in host populations (Nieberding and Morand 2006). Indeed, specialist parasites such as lice frequently exhibit lower genetic diversity and more pronounced geographic structure compared to their hosts (du Toit et al. 2013; Li et al. 2014; Bothma et al. 2021; Pasinelli 2022). Specialist species also have fewer opportunities for dispersal since they only disperse on one host species (and are aggregated within the host population), and this will often result in greater genetic differentiation among different geographic sampling populations (Matthee et al. 2018; Matthee 2020). It is important to note that not all host-specialist parasite systems follow these expectations. Some specialist parasites have demonstrated higher genetic diversity and less pronounced genetic structure compared to generalist parasites from the same host group (Titus and Daly 2017; Sweet and Johnson 2018; Matthews et al. 2023).

To test the hypothesis that a permanent species-specific louse species will show more phylogeographic structure than a host species, we investigated the population genetics of a parasitic louse species, *Johnsonpthirus heliosciuri*, and its rodent squirrel host, *Paraxerus cepapi* (Smith’s bush squirrel), in South Africa. The squirrel is the most abundant diurnal tree squirrel throughout Sub-Saharan Africa and is generally found in savannah woodland (Kingdon 1997; Monadjem et al. 2015). They nest in small family groups of approximately five individuals, and these nests are normally in old, hollowed branches of mopane (*Colophospermum mopane*) and *Vachelia* (previously known as *Acacia*) trees (Linzey and Kesner 1997; Skinner and Chimimba 2005). It has been proposed that *P. cepapi* is restricted in its dispersal predominantly due to habitat fragmentation (Power and Child 2016), which also appears to be the leading threat to squirrel dispersal globally (Gurnell and Pepper 1993; Wauters et al. 1994; Delin & Andrén, 1999). Information regarding the landscape configuration in which *P*. *cepapi* occurs is limited. However, Munyati and Kabanda (2009) made use of an integrated dataset consisting of Landsat images from 1990, 2000 and 2006, alongside municipal demographic data to assess changes in forest and woodland landscapes in the Limpopo province of South Africa. The results indicated a 20% loss in forest and woodland cover over the 16-year period primarily due to urban expansion, agricultural development and the overharvesting of trees for fuelwood (Department of Environmental Affairs and Tourism 2003). To date, no phylogeographic research has been done on *P. cepapi*, but based on phylogeographic analyses done on other squirrel species elsewhere across the globe (Dozières et al. 2012; Liu et al. 2014; Brandler et al. 2021), it is predicted that over small geographic ranges, Smith’s bush squirrel will show limited geographic structure when compared to the louse species.

*Johnsonpthirus heliosciuri* is only one of four sucking louse species known to infest *P. cepapi* (Johnson 1960; Viljoen 1977; Durden and Musser 1994). *Werneckia paraxeri* was recorded on an unknown number of *P. cepapi* individuals in Namibia (Johnson 1960), and *Enderleinellus heliosciuri* and *Johnsonpthirus suahelicus* were recorded on an unknown number of *P. cepapi* individuals at undisclosed localities (Durden and Musser 1994). Of these, *J. heliosciuri* is the most prevalent species on Smith’s bush squirrel in South Africa (Raubenheimer et al. 2025; submitted), and given the documented intimate relationship between *P. cepapi* and *J. heliosciuri*, it is hypothesized that the parasite will show pronounced phylogeographic structure when compared to *P. cepapi* (Liu et al. 2014; du Toit et al. 2013; Matthee et al. 2018; Sweet et al. 2018; Bothma et al. 2021). Should the parasite show genetic structure in the absence of structure in the host, the louse population genetics may thus act as a biological magnifying glass and as a proxy for fine-scale geographic movement of the host individuals (Nieberding et al. 2004; Nieberding and Morand 2006).

## Materials and methods

### Study area and sample collection

**Fig. 1:**
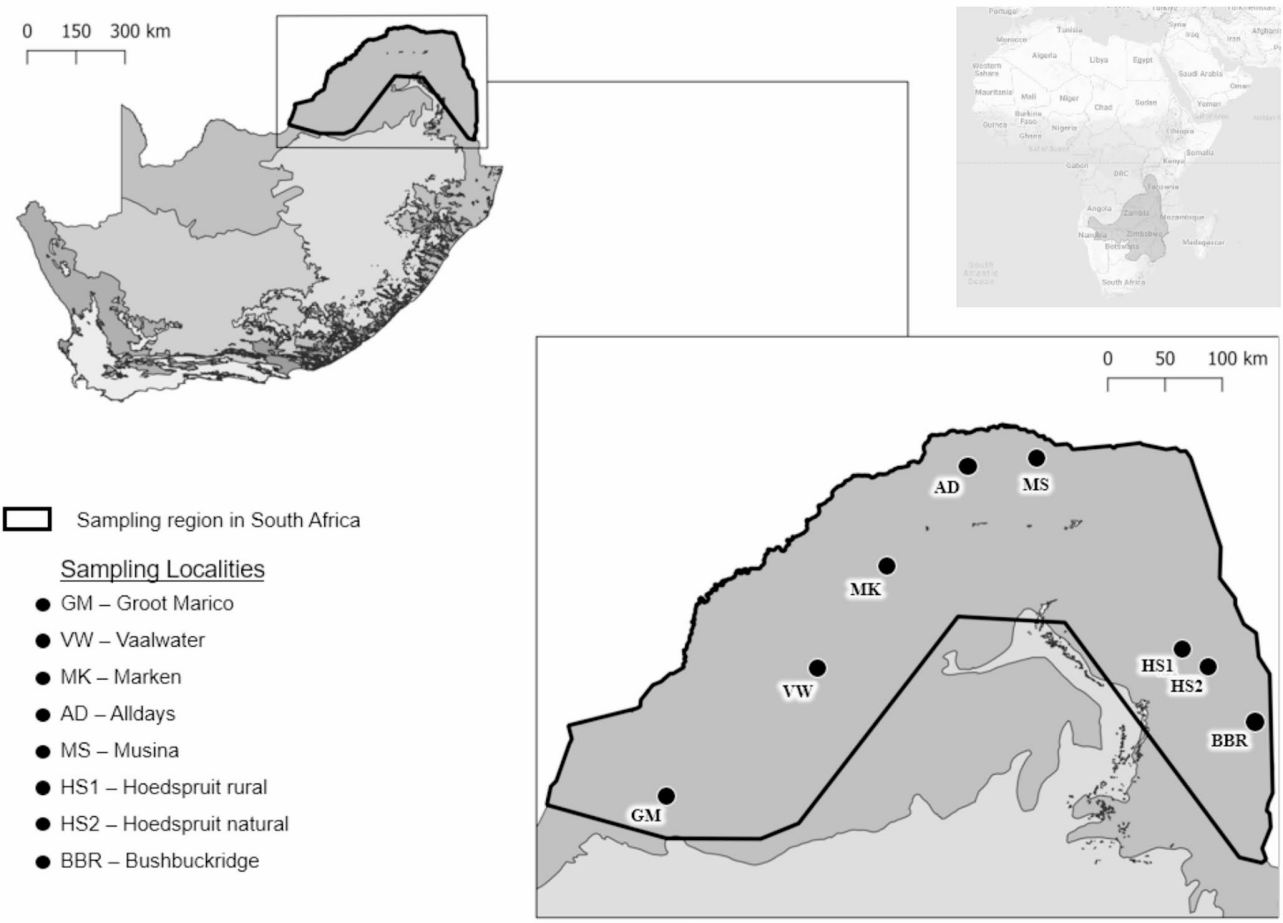
Eight localities where *P. cepapi* were obtained in the Savanna biome, South Africa. The various biomes of South Africa are indicated by the different shades of grey and obtained from openAFRICA (2015). The bold black outline represents the documented distribution range of the squirrel in South Africa. Sampling locality codes correspond to Table 1. The inset map indicates *P. cepapi*’s entire range in Sub-Saharan Africa

*Paraxerus cepapi* individuals were opportunistically sampled through roadkills and culling throughout the species’ range in South Africa, which spans the Savanna woodlands of Limpopo, Mpumalanga and the North West provinces (Table 1; Fig. 1). Due to the opportunistic sampling method used in this study, sample sizes unfortunately varied greatly among sampling sites (Table 1). All carcasses were placed in individual plastic bags and frozen.

**Table 1:** Sampling localities in the savanna biome of South Africa (2020–2024) indicating abbreviation codes, geographic coordinates, the number of *P. cepapi* and *J. heliosciuri* sequenced and louse prevalence

Locality	Code	Geographic coordinates	Number of hosts sequenced (control region)	Number of hosts sequenced (EEF1a1)	J. heliosciuri prevalence (%)	Number of lice sequenced (COI)	Number of lice sequenced (EEF1a1)
Groot Marico	GM	25˚35’5,87’’S 26˚24’24,87’’E	2	3	66.67	4	3
Vaalwater	VW	24˚29’6,04’’S 27˚48’51.63’’E	2	2	100	2	0
Marken	MK	23˚35’20’’S 28˚23’6.82’’E	8	8	30.77	7	5
Alldays	AD	22˚41’56.93’’S 29˚6’1.57’’E	6	6	71.43	4	2
Musina	MS	22˚22’19.23’’S 29˚57’35.34’’E	4	4	0	0	0
Hoedspruit rural	HS1	24˚25’41.36’’S 31˚10’22.96’’E	10	6	95	10	8
Hoedspruit natural	HS2	24˚20’45.96’’S 30˚58’23.88’’E	17	18	73.91	15	15
Bushbuckridge	BBR	24˚55’16.68’’S 31˚33’35.28’’E	2	2	50	1	0
Total			**51**	**49**	**73.40**	**43**	**33**

### Host examination, parasite removal and identification

Carcasses were thawed in the laboratory and lice were systematically removed using fine point forceps and a Leica MZ75 stereomicroscope. All lice were placed in 100% ethanol and morphologically sorted using taxonomic reference keys (Cummings 1912; Ferris 1919; Werneck 1947). Host tongue tissue was removed and stored in 100% ethanol for further analysis.

### DNA extraction, PCR amplification and sequencing

Total genomic DNA was extracted from the hosts and lice using the Nucleospin Tissue kit and following the protocol set out by the manufacturer (Macherey-Nagel, Duren, Germany). After the extraction of DNA from the louse individuals, the exoskeletons were removed and slide mounted in polyvinyl alcohol (PVA) to confirm species identification based on morphological characteristics. To obtain maximum phylogeographic resolution for the host, the non-coding fast evolving mitochondrial DNA (mtDNA) control region was amplified and sequenced while for the parasite, the slower evolving COI region was used. Different gene fragments were used because the slow evolving COI region may be too conservative to obtain any fine scale structure in the host DNA (Dozières et al. 2012; Liu et al. 2014; Brandler et al. 2021) but the same gene marker has been demonstrated to provide resolution for phylogeographic structure of lice over short geographic distances (du Toit et al. 2013; Bothma et al. 2021). The N777 (L15910; Hoelzel et al. 1991) and DLH1 (H16498; Shields and Kocher 1991) primers were used for the control region and for the COI region, the LCO1490 and HCO2198 primers from Folmer et al. (1994) were used. The Eukaryotic Elongation Factor 1 -alpha (EEF1a1) gene was amplified and sequenced for host and louse specimens using EF-1aFor3 and Cho10 primers published by Danforth and Ji (1998).

Amplification followed standard PCR protocols in a GeneAmp^®^ PCR system 2700 thermal cycler (Applied Biosystems) with a total reaction volume of 25 µl. Thermal cycling conditions included an initial denaturation of 5 min at 94 °C, followed by 35 cycles of denaturation for 30 s at 94 °C, annealing for 45 s at temperatures ranging from 46 to 50 °C and extension for 45 s at 72 °C. This process was followed by a final extension period of 5 min at 72 °C. PCR products were visualised on a 1% agarose gel using electrophoresis and fragments that amplified positively were purified and sequenced in the forward direction using BigDye Chemistry. Sequence analyses were done on an automated sequencer (ABI 3730 XL DNA Analyzer, Applied Biosystems) at the Central Analytical Facility on the Stellenbosch Campus.

#### Data analysis

Each sequenced region (mitochondrial and nuclear) was analysed separately but followed similar analyses. At first, the host and louse sequence authenticities were confirmed using the BLASTN tool on Genbank (Nucleotide BLAST: Search nucleotide databases using a nucleotide query). Thereafter sequences were visually inspected for any ambiguous bases and edited using BioEdit sequence alignment editor version 7.7.1 (Hall 2005). The beginning and end of sequences (approximately 20–30 base pairs at each end) were trimmed to avoid the introduction of sequencing error and the inclusion of missing data. Sequences were aligned using the ClustalW multiple alignment function (Thompson et al. 2003) in MEGA X (Kumar et al. 2018). The COI gene sequences were translated to proteins to confirm functionality through the absence of stop codons. Molecular diversity indices including the haplotype diversity (h), nucleotide diversity (π) and the number of haplotypes that were all calculated in DnaSP version 6 (Rozas et al. 2017). For the nuclear sequences, heterozygotic positions (where Y and R were present) were coded as heterozygote alleles. The average sequence divergences among host and parasite taxa sampled at the various localities were respectively calculated in Arlequin version 3.5.2.2 (Excoffier and Lischer 2010).

To visualise phylogeographic relationships among the haplotypes across the landscape, statistical TCS haplotype networks were constructed in POPART version 1.7 (Clement et al. 2000). To test for genetic differentiation across the landscape, a global analysis of molecular variance (AMOVA; Excoffier et al. 2005) was performed in Arlequin version 3.5.2.2. A total of 20 000 permutations were performed to obtain significance values. To test for isolation by distance, a Mantel test was performed in Arlequin version 3.5.2.2 using a matrix based on great circle distance between sampling localities and the genetic distance matrix based on pairwise F_ST_ values obtained from AMOVA in Arlequin version 3.5.2.2. A total of 10 000 permutations were performed to obtain significance values.

## Results

Fifty-one control region and 49 EEF1a1 sequences were generated for *P. cepapi* [Genbank accession control region: PV395483.1-PV395533.1; EEF1a1: PV613159.1-PV613207.1], while 43 COI and 33 EEF1a1 sequences were generated for *J*. *heliosciuri* [GenBank Accession COI: PV476954.1-PV476996.1; EEF1a1: PV776832.1-PV776864.1]. Not all squirrels had lice and thus the parasite numbers were lower (Table 1). *Johnsonpthirus heliosciuri* was present on 73.40% of squirrels (Table 1). Stop codons were absent from all the generated COI sequences. Haplotypic and nucleotide diversity was high in both the host and the parasite (Table 2) and at the nuclear DNA level, *P. cepapi* showed higher nucleotide diversity compared to *J*. *heliosciuri.* Haplotypic diversity for *J*. *heliosciuri* was slightly higher compared to the host (Table 2). The average control region sequences distances among sampling localities for the host ranged from 0.72% to 9.73% while the average COI sequence divergences for the parasite range from 0.79% to 5.27% (Table 3).

**Table 2:** The gene region, total number of individuals sequenced, number of base pairs sequenced, total number of haplotypes, nucleotide and haplotype diversity, and Phi_st_ for the host, *P. cepapi*, and louse, *J. heliosciuri*

	Gene region	Number of individuals sequenced	Base pairs sequenced	Total haplotypes (*n*)	Nucleotide diversity (± SE)	Haplotype diversity (± SE)	Phi_st_
mtDNA							
*Paraxerus*	Control region	51	558	37	0.033 (± 0.006)	0.979 (± 0.010)	**0.304**
*Johnsonpthirus*	COI	43	608	23	0.027 (± 0.002)	0.900 (± 0.038)	**0.797**
nuDNA							
*Paraxerus*	Eef1a1	49	336	24	0.007 (± 0.001)	0.750 (± 0.044)	**0.211**
*Johnsonpthirus*	Eef1a1	33	340	12	0.004 (± 0.0005)	0.777 (± 0.041)	**0.071**

Significant values (P < 0.05) are indicated in bold

**Table 3:** Average percentage MtDNA sequence divergences (*±* SE) among localities for *J. heliosciuri* (above the diagonal) and *P. cepapi* (below the diagonal). Sampling localities are labelled as: (1) Groot marico; (2) Vaalwater, (3) marken, (4) alldays, (5) musina, (6) hoedspruit rural, (7) hoedspruit natural, (8) bushbuckridge. No lice were removed from musina hosts

	1	2		3	4	5		6		7		8
1	****	1.12 (± 0.52)	5.14 (± 1.79)		4.45 (± 1.80)	-	2.62 (± 1.03)		3.04 (± 0.75)		2.25 (± 1.18)	
2	0.92 (± 0.58)	****	5.27 (± 2.36)		4.58 (± 2.30)	-	3.18 (± 1.67)		3.60 (± 1.13)		2.85 (± 1.43)	
3	3.16 (± 1.55)	2.72 (± 1.69)	****		0.84 (± 0.32)	-	4.89 (± 1.60)		5.19 (± 1.13)		4.16 (± 2.36)	
4	7.80 (± 3.32)	7.57 (± 3.71)	9.19 (± 2.71)		****	-	4.20 (± 1.71)		4.50 (± 1.18)		3.45 (± 2.11)	
5	1.54 (± 0.69)	0.81 (± 0.51)	3.14 (± 1.40)		7.85 (± 2.75)	****	-		-		-	
6	1.34 (± 0.50)	0.72 (± 0.30)	3.00 (± 1.31)		7.73 (± 2.52)	1.18 (± 0.43)	****		0.79 (± 0.29)		1.14 (± 0.79)	
7	1.62 (± 0.53)	1.04 (± 0.41)	3.13 (± 1.18)		7.88 (± 2.34)	1.51 (± 0.46)	1.39 (± 0.31)		****		1.59 (± 0.59)	
8	3.50 (± 2.28)	2.95 (± 2.51)	4.45 (± 1.86)		9.73 (± 4.08)	3.32 (± 1.97)	3.32 (± 1.91)		3.02 (± 1.66)		****	

The global analyses of molecular variance for the control region indicated significant (*P* < 0.05) genetic differentiation among *P*. *cepapi* populations but the greatest percentage of variation (69.64%, *P* < 0.05) was observed within populations. Similar results were observed at the nuclear DNA level for *P. cepapi* where most of the variation was again contained within populations (78.91%, *P* < 0.05). In contrast to the host, the AMOVA analyses suggested that at the mtDNA level, *J*. *heliosciuri* was significantly differentiated among populations (79.73%, *P* < 0.05). At the nuclear DNA level, where effective population size of the marker is four times larger, the results for *J*. *heliosciuri* suggested less differentiation among populations and most of the variation within the populations (92.85%, *P* < 0.05). The marked population structure in the parasite was further supported by significant isolation by distance (rY1 = 0.681; *P* < 0.05).

**Fig. 2:**
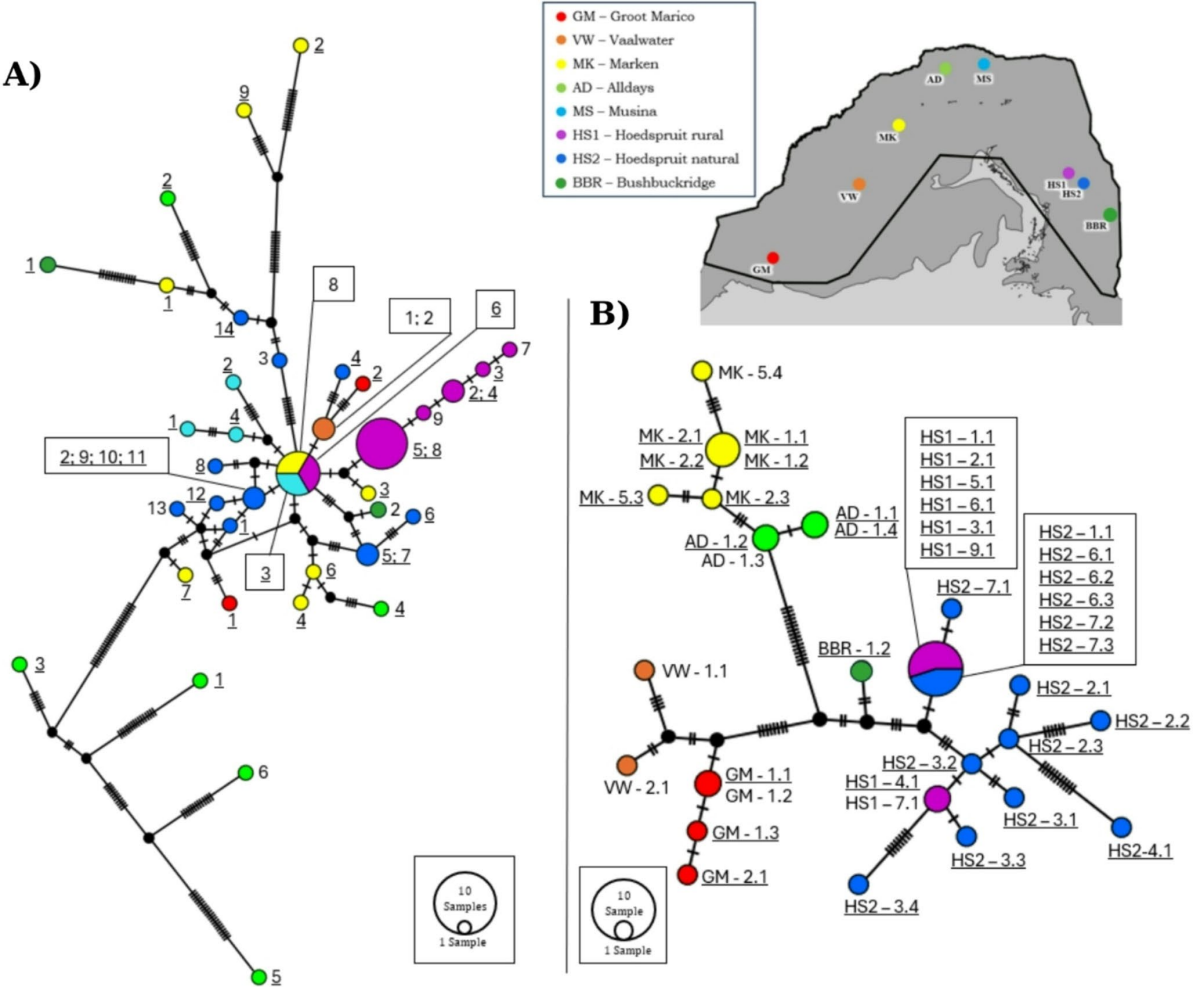
TCS haplotype networks (**A**) *P. cepapi* mtDNA control region (**B**) *J. heliosciuri* mtDNA COI haplotypes. In network **A**, each sample is labelled using a code that indicates its locality (matching the legend), followed by the host number. Hosts codes for individuals whose lice did not successfully amplify were excluded from the network. Numbering in network **A** represent host individual numbers and numbering in **B** are labelled with the locality code, followed by the host number (corresponding to **A**) and louse number. For example, HS2–7.1 refers to Hoedspruit natural host 7, louse 1 (corresponding to **A**). Vertical lines on the branches represent the number of mutations separating haplotypes. The size of the circle is proportional to the number of individuals that share the haplotype. Colours correspond to the different sampling localities on the map. The map is an inset of Fig. 1

**Fig. 3:**
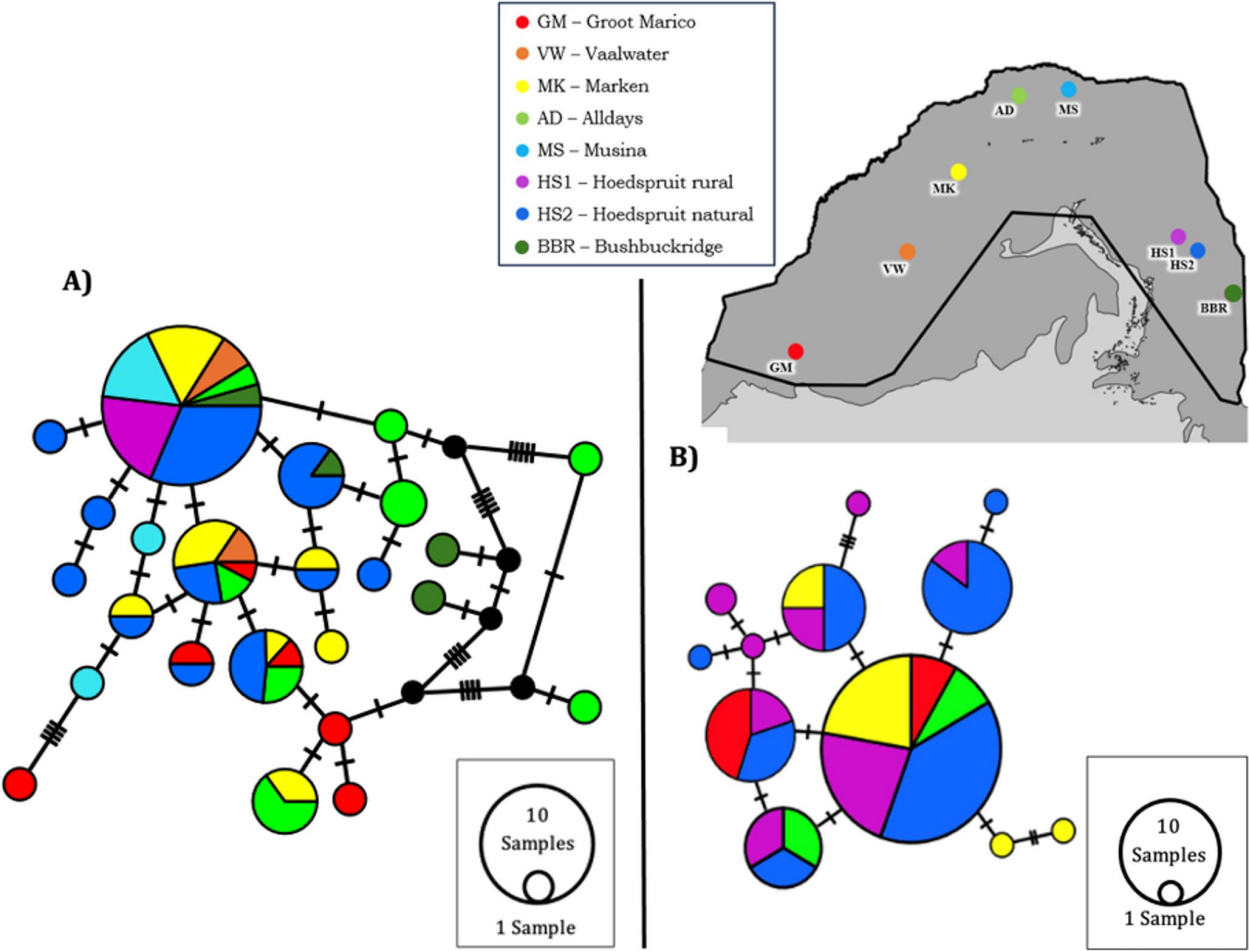
TCS haplotype networks (**A**) *Paraxerus cepapi* nuDNA haplotypes (**B**) *Johnsonpthirus heliosciuri* nuDNA haplotypes. Vertical lines on the branches represent the number of mutations separating haplotypes. The size of the circle is proportional to the number of individuals that share the haplotype. Colours correspond to the different sampling localities on the map. The map is an inset of Fig. 1

A TCS haplotype network revealed no clear geographically structured groups based on the large number of unique control region mtDNA haplotypes (*n* = 31) for *P. cepapi* (Fig. 2A). The network mostly exhibited a complex topology with a few common shared and a large number of unique haplotypes across the geographic range (Fig. 2A). The COI mtDNA TCS network for *J*. *heliosciuri* revealed geographically structured haplo-groups with 17 unique haplotypes (Fig. 2B). Based on the number of mutational steps separating haplotypes, the diverse haplotypes of the geographically close Hoedspruit rural and Hoedspruit natural localities grouped closer together (Fig. 2B). Geographically close Marken and Alldays individuals have fewer mutational steps separating them when compared to other localities and likewise Vaalwater and Groot Marico show a closer evolutionary relationship (Fig. 2B). At the nuclear DNA level, 15 unique haplotypes were recorded for *P. cepapi* and a large number of shared alleles were observed in the TCS network (Fig. 3A). One common allele was shared among seven of the eight sampling localities (Fig. 3B). The nuclear DNA TCS network for *J*. *heliosciuri* presented six unique haplotypes and a large amount of allele sharing between sampling localities and most of the alleles were separated by one mutational step (Fig. 3B).

## Discussion

Population genetic studies on squirrels and their associated parasites are not widely available. Most previous studies investigating squirrel phylogeography reported significant variation among populations sampled across large geographic areas, typically attributing this to geographic distance among sampling sites and/or barriers to dispersal (Aghbolaghi et al. 2019; Rocha et al. 2022). In this study, sampled over a small geographic area, no obvious geographic barrier to dispersal exists for *P. cepapi*, and the vast majority of genetic diversity was contained within populations.

The lack of geographic structure among sampling sites in the host, *P. cepapi* (despite using a faster-evolving mitochondrial marker), and the clear geographic structure observed in *J. heliosciuri* (based on a slower-evolving marker), was the most striking result of this study. In the parasite, most closely related haplotypes clustered by sampling site, and haplotypes from geographically proximate locations grouped together, as also reflected in the significant isolation by distance result. The high level of differentiation among parasite sampling sites was further supported by the global AMOVA analysis, where 79.73% (*P* < 0.05) of the variation was attributed to differences among geographic sampling sites. In contrast, only 7.15% (*P* < 0.05) of the variation in the squirrel host was explained by differences among populations. This supports the well-established notion that parasites often exhibit greater phylogeographic structure than their hosts due to a higher evolutionary rate, smaller effective population sizes, and limited dispersal capacity (Johnson et al. 2003; McCoy et al. 2003; Criscione et al. 2005; Martinu et al. 2018).

The lack of population structure in *P. cepapi* and the strong structure present in *J. heliosciuri* is, however, rarely reported in the literature and contradicts expectations for permanent, species-specific parasites (Hafner and Page 1995; Whiteman et al. 2007; Light and Hafner 2008; du Toit et al. 2013; Bell et al. 2021). This incongruence was observed only in the mtDNA data, while the nuclear DNA data did not reflect the same pattern, likely due to the absence of a phylogenetic signal for the nuclear marker used. Parallel evolutionary patterns between parasite and host are typically facilitated by high host specificity, direct life cycles, small effective population sizes, and vertical transmission (Nadler 1995; Clayton et al. 2003; Johnson et al. 2003; McCoy et al. 2003; Criscione et al. 2005; Kim 2006). However, host vagility is equally important, since hosts with high dispersal potential will exhibit reduced genetic structure in their parasites, while hosts with low dispersal ability tend to show more genetic structuring among parasite populations (Matthee et al. 2018). If the latter scenario applies, it suggests that Smith’s bush squirrel may be more movement-restricted across localities than previously expected, which aligns with other large-scale studies on squirrels globally (Dozières et al. 2012; Liu et al. 2014; Aghbolaghi et al. 2019; Rocha et al. 2022).

The question remains: why does analysis of the fast-evolving mtDNA molecule fail to reveal phylogeographic structure in the host, while such structure is evident only in the mtDNA of the permanent parasitic louse? High host specificity (which increases the likelihood of lineage extinction if the host becomes locally extinct), limited dispersal ability (body contact is needed among host individuals), and the parasite’s small effective population size all contribute to a higher fixation rate of mtDNA differences at specific localities. Reduced gene flow leads to stronger genetic drift within populations (Criscione et al. 2005; Nieberding and Morand 2006; Stefka and Hypša 2008). Geneflow among localities are further hampered by louse prevalence since lice were not detected on all squirrels. Should *P*. *cepapi* individuals from these low prevalence areas disperse without carrying the louse, host movement would not facilitate louse gene flow, further contributing to the pattern observed within *J*. *heliosciuri*. The nuclear data did not show the same pattern, as expected, since nuclear markers typically evolve more slowly and have an effective population size four times larger than that of mtDNA (Brown et al. 1979; Zhang and Hewitt 2003; Chan et al. 2021). These factors often render single-copy nuclear genes insufficiently sensitive to detect intraspecific divergence (also see du Toit et al. 2013; Bothma et al. 2021).

The pattern of shared haplotypes among sampling sites in *P. cepapi*, the high level of genetic diversity, and the confinement of nearly all variation within populations are commonly interpreted as evidence of high gene flow among localities (Hale et al. 2001; Brandler et al. 2021; Fusco et al. 2023). In contrast, the mtDNA genetic structure of *J. heliosciuri* in this study suggests the opposite. If this holds, the parasite indeed serves as a proxy and magnifying glass for current connectivity among squirrel populations (Nieberding et al. 2004; Nieberding and Morand 2006). The lack of phylogeographic structure in the host is likely linked to recent habitat fragmentation (Munyati and Kabanda 2009; Power and Child 2016) and the inability of mtDNA to detect such recent events, even when using the control region. Species-specific parasites such as lice, on the other hand, are known to be more sensitive to stochastic evolutionary events and can provide a more refined proxy for evolutionary processes in the host (Nieberding et al. 2004; Nieberding and Morand 2006). Based on these findings, human-induced habitat fragmentation may indeed be limiting squirrel movement across the landscape (Munyati and Kabanda 2009; Power and Child 2016), and such anthropogenic effects are too recent to be captured in the squirrel’s mtDNA structure. The large number of shared haplotypes is thus best explained by ancestral polymorphism (Maddison and Knowles 2006), rather than by high current levels of gene flow in *P. cepapi*.

Our study provide evidence that parasites can indeed be useful in conservation since in this study they serve as an indicators for host connectivity (Gagne et al. 2022). To fully understand the system and better assess connectivity among Smith’s bush squirrel populations across Africa, future work should include larger sample sizes, more genetic markers, and broader geographic sampling.

## Data Availability

Fifty-one control region and 49 EEF1a1 sequences were generated for P. cepapi [Genbank accession control region: PV395483.1-PV395533.1; EEF1a1: PV613159.1-PV613207.1], while 43 COI and 33 EEF1a1 sequences were generated for J. heliosciuri [GenBank Accession COI: PV476954.1-PV476996.1; EEF1a1: PV776832.1-PV776864.1].
